# Influence of Voxelotor–hemoglobin complexes in the estimation of hemoglobin S levels by the current standard of care laboratory evaluation techniques

**DOI:** 10.3389/fmed.2023.1149281

**Published:** 2023-04-20

**Authors:** Salam Alkindi, Ahmed Al Subhi, Abubakr E. H. Ali, Anil V. Pathare

**Affiliations:** ^1^Department of Hematology, Sultan Qaboos University Hospital, Muscat, Oman; ^2^College of Medicine & Health Sciences, Muscat, Oman

**Keywords:** sickle cell disease, Voxelotor, high performance liquid chromatography, exchange transfusions, hemoglobin D Punjab

## Abstract

**Background:**

Sickle cell disease is an inherited disorder characterized by the presence of sickle hemoglobin (HbS). The process of Hb molecule polymerization is a pivotal step in the sickling process. Voxelotor, a recently approved novel therapeutic agent, is known to interfere with polymerization. We aim to study the impact of Voxelotor on Hb variants analysis using high performance liquid chromatography (HPLC).

**Material and methods:**

We are reporting the impact of Voxelotor on Hb variants analysis using HPLC after an informed consent and medical research committee approval. Data was collected from eight patients who are enrolled in the GBT440-034OL study using electronic medical records, to evaluate the Hb levels, hemolytic markers and the clinical response.

**Results:**

Our patients were well-balanced for gender, with a mean age of 31.1 years (19–50). Six patients showed a significant improvement in the Hb level, with reduced reticulocytes, bilirubin, LDH and an improved clinical outcome. Interestingly, these patients showed the appearance of a split band of Hb S and D on HPLC impacting significantly on HbS level. Two patients did not show any improvement on laboratory parameters, and no changes on their HPLC analysis.

**Conclusions:**

We report here eight patients on Voxelotor therapy, six of which showed improved hemolytic markers and anemia and demonstrated the appearance of HbD peak on the HPLC chromatogram. Therefore, the absence of HbD on HPLC or other laboratory methods for estimating HbS in patients on Voxelotor therapy, gives the clinician a possible hint regarding the patient's compliance with the drug.

## Introduction

Sickle cell disease (SCD) is an inherited disorder characterized by the presence of hemoglobin (Hb) S. It manifests as a homozygous state (HbS/S) or double heterozygous state, when HbS is co-inherited with other Hb variants such as β-thalassemia, Hemoglobin C (HbC), Hemoglobin D-Punjab (HbD Punjab), Hemoglobin E (HbE), and others (1). SCD results from a point mutation involving the β-globin chain of hemoglobin, where glutamic acid is replaced with valine (βGlu → Val). At low oxygen tension, hemoglobin molecule undergoes a process of polymerization, which is thought to play a critical role in the process of sickling.

Hydroxyurea (HU) was the first approved disease-modifying intervention in adults and children with SCD; however, the introduction of L-glutamine, Crizanlizumab and Voxelotor provides an additional approved treatment options for the management of SCD complications (2).

The US FDA has approved Voxelotor for treating adults and children (≥4 years of age) with SCD based on the results from the HOPE and HOPE-KIDs trials, where it demonstrated an improved Hb level, and reduced hemolysis, with an improved rate of vaso-oclusive crises (VOCs) (3, 4). Post-licensure of Voxelotor, real-world studies in the US (5) and the EU (6) concur with the HOPE studies' result. Voxelotor brings its effects by reversibly binding to, and stabilizing HbS in an oxygenated state to ultimately inhibit the polymerization of HbS and increase oxygen affinity in RBCs, thereby preventing red blood cells from sickling. Assessment and monitoring of the level of HbF and Hb S are desirable and necessary in many situations including monitoring the response to hydroxyurea (measurement of Hb F level) and also in assessing the effectiveness of red cell exchange (measurement of Hb S) procedures. Although the addition of Voxelotor therapy to the drugs already available for SCD patients is highly desirable, physicians and scientists should be aware of its interference with laboratory tests that are routinely performed to measure HbS levels, and other Hb Variants.

Voxelotor seems to be a potent inhibitor of HbS polymerization *in vitro* as well as *in vivo* animal models in SCD individuals, but *in vitro* spiking experiments showed that Voxelotor could modify multiple variants and sub-variants of hemoglobins, including HbA, HbS, HbC, HbD-Punjab, HbE, HbA2, and HbF (7). Mass Spectrophotometric studies using matrix-assisted laser desorption/ionization (MALDI) confirmed that Voxelotor can bind with the N-terminus of hemoglobin alpha chain forming hemoglobin S-Voxelotor complexes (8). Consequently, these HbS-Voxelotor complexes could lead to inconsistencies in HbS quantification by the currently used standard of care laboratory evaluations techniques, including high-performance liquid chromatography (HPLC), capillary zone electrophoresis (CZE) and isoelectric focusing techniques (IEF) (7, 8). Thus, the question arises as to how do we monitor HbS in patients on Voxelotor therapy that may require blood or exchange transfusion, where the actual percentage of HbS plays a significant role in their management. Here we are reporting the impact of Voxelotor on various Hb Variants, and we also hypothesize that the appearance of abnormal Hb variant on HPLC may reflect patient compliance with the drug.

## Materials and methods

We report our experience managing eight SCD patients receiving Voxelotor. Results were obtained from the electronic medical records using the data from the pre-and post-Voxelotor therapy periods, including Hb levels, biochemical markers of hemolysis, and results of Hb variant analysis using HPLC as our laboratory performs HbS quantitation by HPLC using the BioRad Variant II β-thalassemia short program (Bio-Rad Laboratories, Hercules, CA, USA). We also analyzed the impact of the various genotypes that are available in our cohort and the impact of Voxelotor therapy on the analysis. The patients are enrolled on the ongoing open label GBT440-034 OL study which was approved by the medical ethics committee (SQU-EC/043/18).

## Results

We analyzed data from eight patients who were enrolled in this study. Our eight cases, showed a 1:1 male: female distribution, with a mean age of 31.1 years (19–50). Table 1 displays the SCD-significant laboratory parameters including hemoglobin (Hb), retics, lactate dehydrogenase (LDH), bilirubin, HbS and HbD. Across the eight subjects, there is a significant increase in Hb levels (*P* = 0.02, Wilcoxon signed ranks test) and a significant decrease in retics (*P* = 0.03), LDH (*P* = 0.01), bilirubin (*P* = 0.02) and HbS (*P* = 0.0001). Interestingly HbD variant was increased from zero to a mean of 23.7% post-Voxelotor dose due to forming HbS-Voxelotor complexes that run in the HbD window in HPLC. Cases 7 and 8 showed no change in Hb, or other laboratory markers, and no appearance of HbD variant on their HPLC. The results also show a significant reduction (*P* < 0.05) of the hemolytic parameters including reticulocytes, LDH, and bilirubin. Expectedly, this corresponded to a significant reduction in recurrent VOC episodes reported by these patients.

**Table 1:** Demography and laboratory parameters in SCD cohort on Voxelotor.

**Voxelotor cohort**	**Demography**		**Hb g/dl**		**Retics %**		**LDH** μ**/L**		**Bilirubin mg/dl**		**HbS %**		**HbD %**	
	**Sex**	**Age**	**Pre**	**Post**	**Pre**	**Post**	**Pre**	**Post**	**Pre**	**Post**	**Pre**	**Post**	**Pre**	**Post**
Case 1	M	35	7.8	9.6	13.7	3.5	1,137	303	87	20	92.8	73.5	0	22
Case 2	M	40	8.8	13.3	4.1	2.7	1,659	232	70	13	87.1	56.4	0	36.1
Case 3	F	25	7.1	10.8	15.7	8	492	282	59	18	85.5	42.4	0	52.1
Case 4	M	19	10.4	16.1	5	1.3	1,059	185	53	40	87.5	56.8	0	36.1
Case 5	F	26	7.7	13.2	6.3	2.4	419	343	18	9	91.2	56.2	0	28.5
Case 6	M	33	7.8	11.6	12	6.2	662	392	106	61	86.1	46.8	0	15.1
Case 7	F	50	7.1	6.7	9.4	7.6	539	497	33	21	89.6	47.8	0	0
Case 8	F	21	8.5	7.9	8	9.3	391	408	64	54	93.9	92.4	0	0
Mean		31.1	8.2	11.2	9.3	5.1	795	330	61.3	29.5	89.2	59	0	23.7
+ SD		9.7	1.0	2.9	3.9	2.8	420	94	26.2	18.3	2.9	15.3	0	17.1
Min		19	7.1	6.7	4.1	1.3	391	185	18	9	85.5	42.4	0	0
Max		50	10.4	16.1	15.7	9.3	1,659	497	106	61	93.9	92.4	0	52.1
Median		29.5	7.8	11.2	8.7	4.9	600	323	61.5	20.5	88.6	56.3	0	25.3
25th		22	7.3	8.3	5.3	2.5	437	245	38	14.3	86.4	47.1	0	3.8
75th		38.75	8.7	13.3	13.3	7.9	1,117	404	82.8	50.5	92.4	69.3	0	36.1
*P*-value			0.02^#^	0.03^#^	0.01^#^	0.02^#^	0.0001^#^	0.002^#^

^#^P < 0.05 vs. pre-dose is considered significant as determined by Wilcoxon signed ranks test. n = 8.

**Figure 1:**
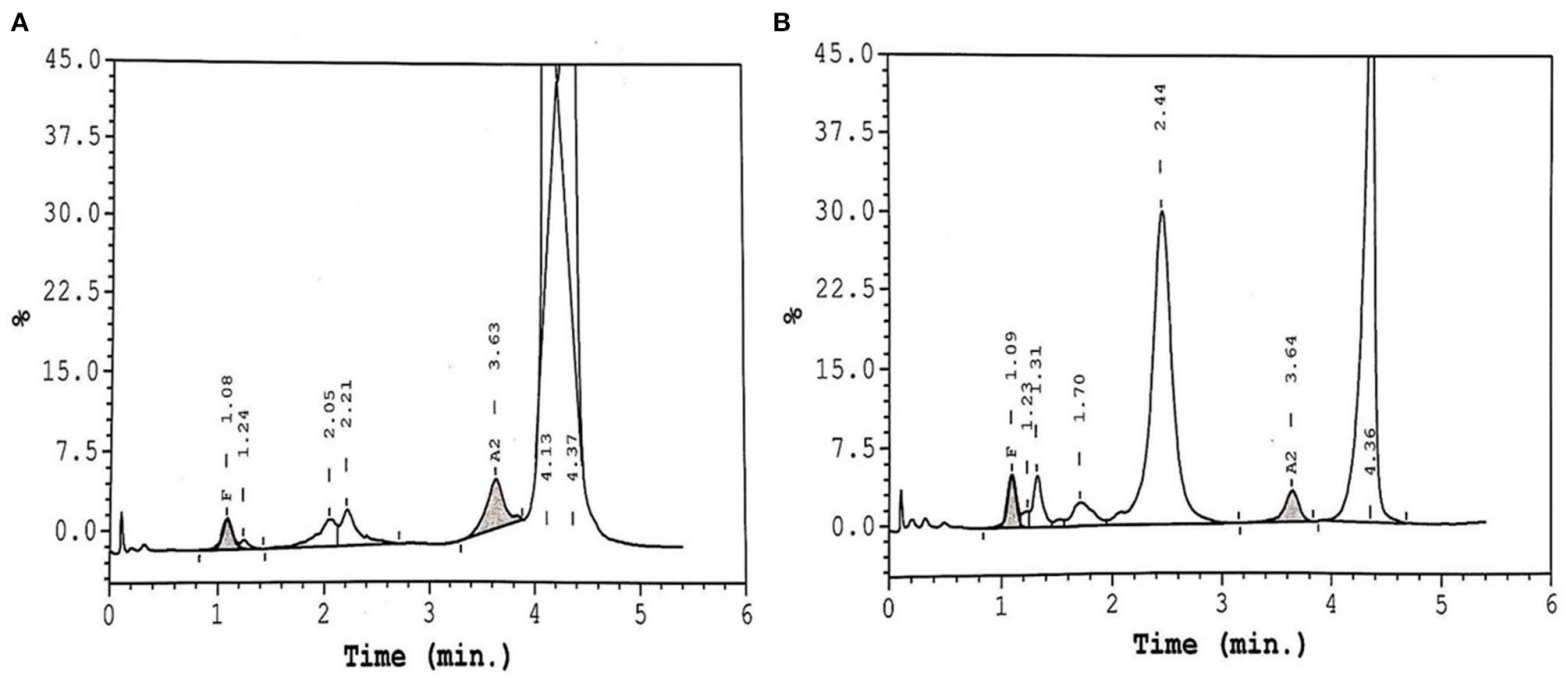
HPLC chromatogram of two patients on Voxelotor therapy. **(A)** Shows the presence of HbS and Hb-Voxelotor fraction in the HbD window. **(B)** Shows a SCD patient on Voxelotor therapy showing only HbS without any HbD peak.

Table 2 shows the different changes in Hb variants HbS, HbF, HbA2, and HbD before and after Voxelotor therapy across the three genotypes commonly seen in our population namely, homozygous HbS/S, and double heterozygous HbS/β0 and HbS/D-Punjab. The patient with HbS/S displayed a decrease in HbS post-treatment, roughly compensated in HPLC by an increase in HbD. Unfortunately, Voxelotor therapy on this patient was interrupted for 2 weeks, and a blood exchange was administered, which affected the results of the HPLC by dramatically decreasing HbS, disappearance of HbD and spike in HbA. Further, patient with HbS/β0 genotype had the highest increase in HbD. Upon reviewing the patient's HPLC chromatograms, we observed a split peak spanning the HbD (retention time 4.10 min) and HbS (retention time 4.37) windows, as displayed in Figure 1A. The rise in the HbD variant is compensated by a reduced HbS, with limited impact on HbF and HbA2, as indicated in Tables 1, 2. We also noted that two of our patients (Cases 7 and 8) did not show any change in their hematological parameters and, as seen on in Figure 1B, and displayed no D-peak on the HPLC test.

**Table 2:** The Impact of Voxelotor on various Hb variants in different genotypes.

**Hb variant**	**Pre Voxelotor**				**Post Voxelotor**				**Voxelotor interrupted**				
	**HbS %**	**HbF %**	**HbA2 %**	**HbD %**	**HbS %**	**HbF %**	**HbA2 %**	**HbD %**	**HbS%**	**HbF%**	**HbA2%**	**HbD%**	**HbA%**
HbS/S	92.8	3.3	3.8	0	73.5	1.1	3.5	22	25.8	1.0	3.2	0	68.8
Hb S/β^0^	87.5	6.3	5.5	0	56.8	3	4.1	53					
HbS/D Punjab^*^	43.7	5.4	3.4	47.5	20	3.0	3.0	67.7					

^*^The patient with genotype HbS/D-Punjab is based on a live case, but is not part of the Voxelotor study; therefore, the post-Voxelotor levels of Hb variants are a rough estimates only.

## Discussion

We present results seen from the data on eight patients with SCD, who are exposed to Voxelotor therapy and they unequivocally show a significant improvement in Hb. This was also correlated with a significant improvement of the hemolytic markers including a significant reduction of reticulocytes, bilirubin and LDH. In HbS/S patients with sickle cell anemia (SCA), there are two peaks, one representing HbS and the other Hb-Voxelotor fraction, the latter having similar mobility as native HbD in HPLC assays. This implies that HPLC could indeed be additionally used to monitor patients' compliance and adherence to the drug. Similarly, we observed an apparent disappearance of the HbD peak upon a 2-week interruption of the medications, due to an acute sickle cell crisis, as illustrated by one of the patients in Table 2, indicating the reversible nature of binding between Voxelotor and hemoglobin. In addition, this patient required a blood transfusion/exchange, as characterized by the discrepancy in the levels of HbS seen in Table 2. Our data also suggested that the impact of Voxelotor on both genotypes HbS/S and HbS/β was roughly identical; however, patient with HbS/β0 genotype had the highest increase in HbD, possibly indicating an enhanced drug effect and binding of Voxelotor to hemoglobin. We also generally observed the higher the rise in Hb, the more pronounced level of HbD on HPLC. Similarly, although we did not have any HbS/D-Punjab patient taking Voxelotor in this group, the HPLC profiles of these patients will be of interest and may cause further confusion when calculating the actual amount of HbS with HbS-Voxelotor complexes in these samples as indicated in Table 2. Therefore, we wish to highlight and emphasize to the laboratory scientists, transfusion medicine specialists, and clinicians providing care for patients with SCD that they need to understand the precise underlying mechanism of Voxelotor therapy and consider its effects on hemoglobin quantitation by existing techniques of hemoglobin estimation.

For this purpose, the laboratory scientist and technicians could easily be trained to recognize the distinct appearance of Voxelotor-modified HbS peaks that run as HbD, as shown in Figure 1, to avoid this pitfall. Further, there is a necessity for appropriate communication between the laboratory staff and the treating clinician to crucially avoid the misinterpretation of HPLC results. Lastly, the decision to report “total” HbS or HbS without HbS-Voxelotor complexes could lead to a confusion in the number of units of RBCs a patient receives in an exchange transfusion setting and, subsequently, estimate its efficiency. Godbey et al. (9), have also reported on this dilemma in their patients coming to the apheresis clinic for the RBC exchange program, who were also on Voxelotor therapy. Thus, additional studies into the structure-function relationship of the hemoglobin-Voxelotor complexes are necessary to understand how quantitative HbS results should be reported in the presence of this agent. We also observed that two of our patients (Cases 7 and 8) did not show any change in their hematological parameters and, showed no appreciable clinical responses, as seen on in Figure 1B, and displayed no D-peak on the HPLC test, possibly indicating that these patients may not be compliant with the drug, although other mechanism for lack of response is also possible. On direct interrogation of these two patients, they also admitted to non-compliance with the drug due to various reasons including drug related side effects.

In conclusion, we reported here eight patients on Voxelotor therapy, six of whom showed improved hemolytic markers and anemia and demonstrated the appearance of HbD peak on the HPLC chromatogram. Therefore, the absence of HbD on HPLC or other laboratory methods for estimating HbS in patients on Voxelotor therapy, gives the clinician a possible hint regarding the patient's compliance with the drug.

## Data availability statement

The raw data supporting the conclusions of this article will be made available by the authors, without undue reservation.

## Ethics statement

The studies involving human participants were reviewed and approved by Medical Research Ethical Committee, College of Medicine & Health Sciences. The patients/participants provided their written informed consent to participate in this study.

## Author contributions

SA and AP were fully involved in the conception and design of the study, acquisition, analysis, interpretation of data, and drafting of the article and critical appraisal before submission. All authors have made substantial contributions and have seen and approved the final version of the manuscript.
